# Full Mouth Rehabilitation Using Telescopic Removable Prosthesis

**DOI:** 10.1155/2022/9536443

**Published:** 2022-10-17

**Authors:** Sara Hussain Alhammadi

**Affiliations:** Dubai Health Authority, , Dubai, UAE

## Abstract

A female, aged 55 years, was presented to the clinic concerned with her inability to function properly due to missing teeth and collapse of her vertical dimension. On examination, remaining tooth position of the upper arch was favorable for telescopic denture and remaining tooth position of the lower arch was favorable for removable prosthesis. The patient requested an aesthetic maxillary and mandibular denture with no visible metal clasps on smiling. Therefore, upper telescopic removable complete denture and lower chrome cobalt removable prostheses were suggested to the patient as conservative treatment to reestablish her occlusal vertical dimension and restore her aesthetics and function.

## 1. Introduction

Prosthodontics plays an essential role in restorative dentistry as the primary objective in dental care is to maintain and preserve the natural dentitions as long as possible to support function. Despite the advances in dental technology, tooth extraction is one of the most widely performed procedures due to multiple reasons. Dental caries was the main reason for tooth extraction followed by periodontal diseases in United Arab Emirates [1]. The pattern of tooth loss has been assessed in different populations in various countries, and there is an increase in Kennedy class I and class II pattern among Saudi Arabia population [2].

Prosthetic rehabilitation of a partially edentulous patient can be established by using a wide range of treatment options. Most preferred prosthetic approaches are conventional removable partial denture, teeth/implant supported over-dentures, fixed partial dentures, and implant supported fixed or partial dentures. The glossary of prosthodontics terms defines over-denture as a removable partial or complete denture that covers and rests on one or more remaining natural teeth, roots, and/or dental implants [3].

Telescopic copings were initially introduced as retainers for removable partial dentures in the beginnings of the 20th century by Starr [4]. The term telescopic crown is defined as an artificial crown constructed to fit over a coping (framework). The coping can be another crown, a bar, or any other suitable rigid support for the dental prosthesis [3]. They are also known as double crowns, where the crown and sleeve coping are used to connect the remaining dentition either teeth or implants to the denture base. The inner (primary) telescopic coping provides retention and stabilization for the outer (secondary) telescopic crown; additionally, it provides protection from dental caries and thermal irritation [5]. While the outer (secondary) telescopic crown engages, the primary coping provides anchorage and friction [5]. Telescopic dentures indicated when there are few remaining or unfavorably distributed abutment teeth [6], when the abutment teeth need to be crowned due to caries, root canal treatment or poor contour [7], abutment teeth with guarded prognosis [8], advanced periodontal cases [9], oral cancer patient [10], and connecting natural teeth to implants [11].

Telescopic dentures utilizing natural dentition has multiple advantages, such as preservation of the alveolar process, provision of better load transmission, maintenance of sensory feedback, and provision of cross arch stabilization [12]. Furthermore, telescopic removable prostheses provide good retention and stabilization, secondary splinting action, directing the occlusal forces along the long axes of the abutments, establish a common path of insertion, and ease of repair and adjustment when an abutment is lost [6, 7, 10]. The telescopic attachment also allows better accessibility to the abutments and the gingiva allowing an effective home care to maintain good oral hygiene [6, 7, 10]. Studies have reported good patient satisfaction rates and better esthetics with telescopic dentures compared to conventional prostheses [7, 10, 13]. Hence, telescopic prostheses can be used as a modality treatment option for restoring the partially edentulous patients, in which it enhances the maintenance and survival of natural abutments. On the contrary, the telescopic prostheses fabrication is clinically and laboratory demanding procedure and costly, with high rates of technical failures, such as de-cementation, fracture of the artificial teeth, metal framework or the denture base, and periodic follow-up [14, 15].

This study describes the management of partially edentulous and periodontally compromised patient. A maxillary telescopic complete denture employed in metal primary copings and metal framework as secondary coping, and a conventional chrome cobalt mandibular removable partial denture were constructed as a full mouth rehabilitation.

## 2. Case Report

**Figure 1:**
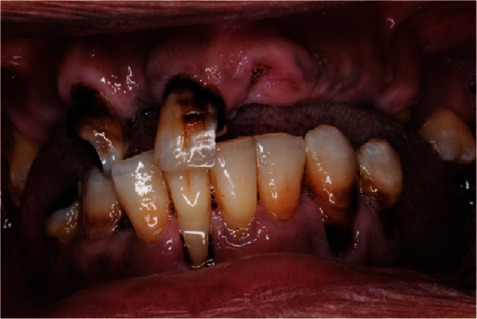
Preoperative frontal view.

**Figure 2:**
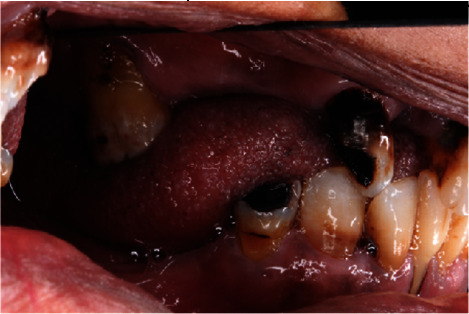
Preoperative right lateral view.

**Figure 3:**
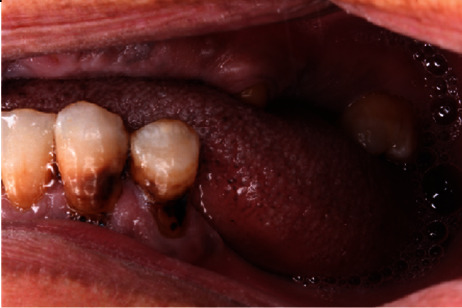
Preoperative left lateral view.

**Figure 4:**
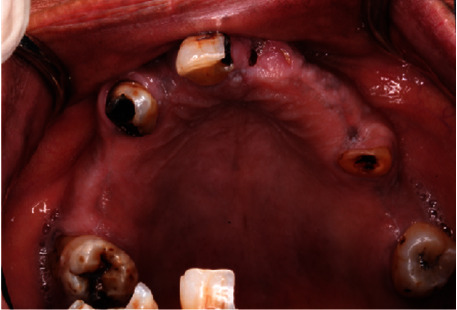
Preoperative upper occlusal view.

**Figure 5:**
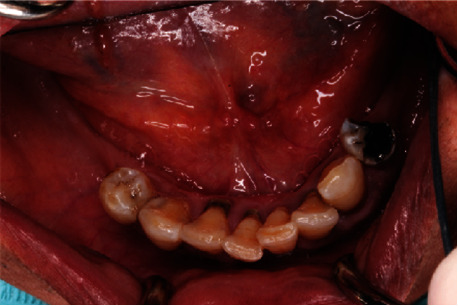
Preoperative lower occlusal view.

**Figure 6:**
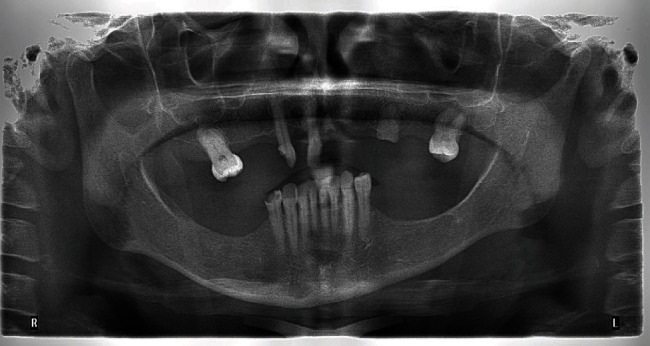
Orthopantomogram radiograph.

A 55-year-old female patient was referred to Prosthodontic Department of Hamdan Bin Mohamed College of Dental Medicine for her inability to chew. After obtaining her medical, dental, and social histories, she was examined clinically and radiographically (Figures 1–6). It was determined that she had lost her teeth due to dental caries and periodontal diseases. Intra oral examination revealed multiple carious lesions (FDI 11, 13, 34, 44, and 45) and (FDI 21 and 24) were remaining roots while (FDI 11, 13, and 45) responded negatively to the pulp sensibility tests hence were diagnosed as necrotic. Periodontal assessment revealed absences of deep pockets and absences of mobility and recession of 5 mm buccally (FDI 16), 2 mm recession labial to (FDI 34), 3 mm recession labial to (FDI 33), and 4 mm recession labial to (FDI 41).

Upper and lower impressions were recorded using irreversible hydrocolloid as a study model. A facebow record was taken, and casts were articulated and mounted in centric relation position on a semi-adjustable articulator (Artex Articulator). Carious teeth (FDI 34 and 44) were restored using composite restoration, while root canal treatment was done for necrotic teeth (FDI 13, 12, and 45), and extraction of the renaming roots (FDI 21, and 24) was done. The plan is to restore the missing teeth using maxillary telescopic complete denture and mandibular conventional chrome cobalt removable partial denture.

## 3. Maxillary Telescopic Complete Denture Fabrication

**Figure 7:**
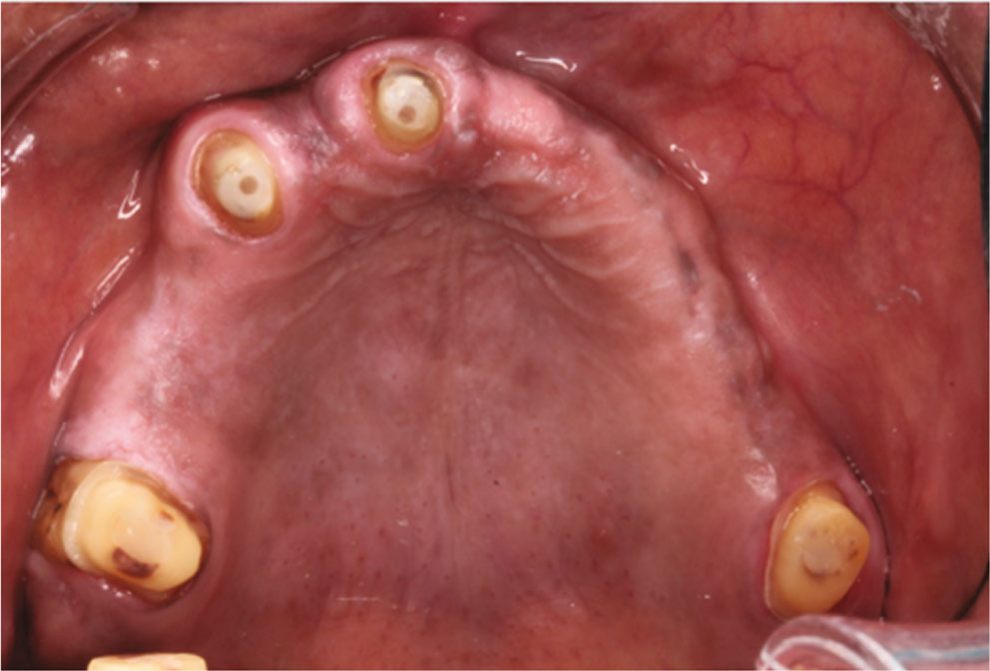
Crown preparation on (FDI 16, 13, 11, and 26).

**Figure 8:**
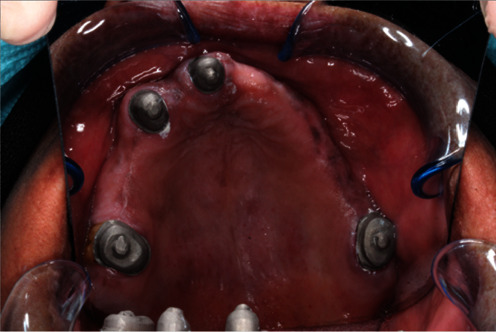
Try-in metal copings in patient mouth (FDI 16, 13, 11, and 26).

**Figure 9:**
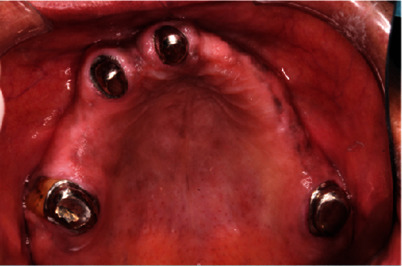
Finished and polished primary metal coping in patient mouth.

**Figure 10:**
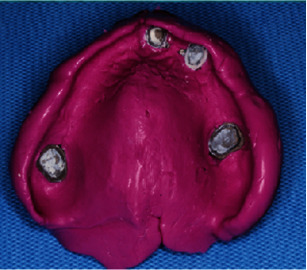
Pick-up impression of primary metal coping.

**Figure 11:**
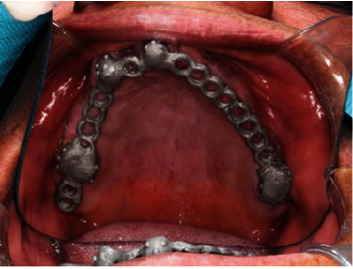
Secondary metal framework.

Root canal treatment was done on the necrotic teeth (FDI 13 and 11), and later was restored using fiber post and composite build up. Crown preparation was done on the abutment teeth on tooth (FDI 16, 13, 11, and 26) to serve as primary coping (Figure 7). Final impression for (FDI 16, 13, 11, and 26) was taken using special tray to provide even thickness of impression material and minimize tissue displacement and dimensional changes of the impression material. Primary semi-precious metal copings were fabricated for (FDI 16, 13, 11, and 26); they were tried in the patient mouth to confirm the setting of the primary copings (Figure 8). Finfishing and polishing is aproceduer in the labratory done to make the primary coping to be smooth and polished. Primary coping and metal framework then were tried in the patient mouth to check for the passive fitting, and again, overall impression was taken utilizing the functional border molding technique (Figures 9–11). The next step is to fabricate occlusal wax bite rim to the jaw relationship records.

## 4. Planning Lower Removable Prostheses Design

**Figure 12:**
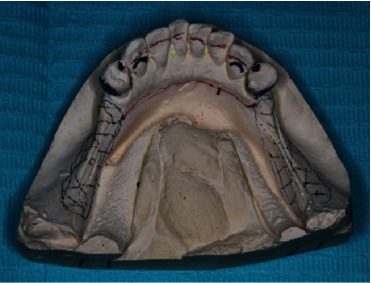
Lower cast with the design for chrome cobalt removable prosthesis.

Lower arch presents Kennedy class I, and lower model was surveyed for fabrication of lower chrome cobalt removable prosthesis and fabrication of survey metal ceramic crown on (FDI 44) (Figure 12). The design included RPA system on the distal abutments (FDI 34 and 44; mesial rest, distal guide plan, and Aker's clasp). Cingulum rests on (FDI 33 and 43) as indirect retention (Figure 12). Lingual plate was used as major connectors as the patient is periodontally compromised and to provide indirect retention. In addition, altered cast technique was used for the distal free end saddle.

## 5. Surveyed Metal Ceramic Crown

**Figure 13:**
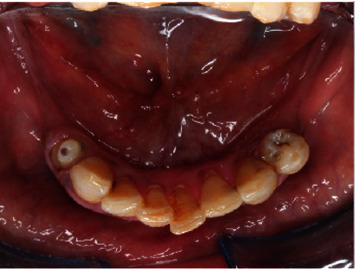
Metal ceramic crown preparation on (FDI 44).

**Figure 14:**
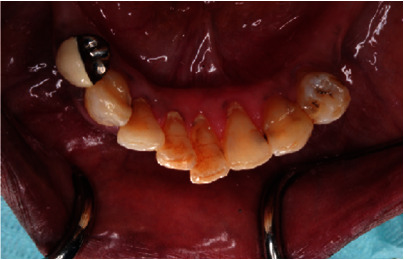
Metal ceramic crown on (FDI 44).

Root canal treatment on (FDI 44) was done and restored with fiber post and composite build up for fabrication. Crown preparation on (FDI 44) was done, and additional tooth preparation on the mesial was done to create additional space for the mesial rest for the chrome cobalt removable prosthesis (Figure 13). Metal ceramic crown was fabricated keeping the lingual and distal aspects in metal and only ceramic layering on the buccal, and it was cemented using resin cement (RelyX™ Unicem), as shown in Figure 14.

## 6. Preparation for Chrome Cobalt Framework

**Figure 15:**
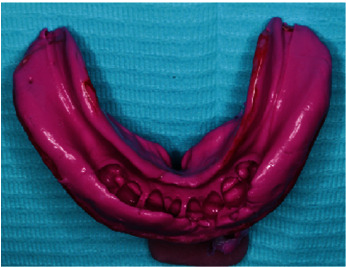
Impression for lower chrome cobalt framework.

**Figure 16:**
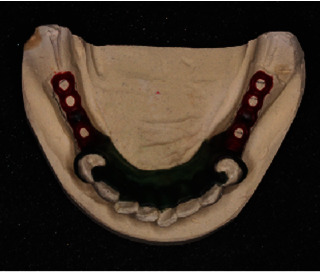
Refractory cast with wax up for chrome cobalt framework.

**Figure 17:**
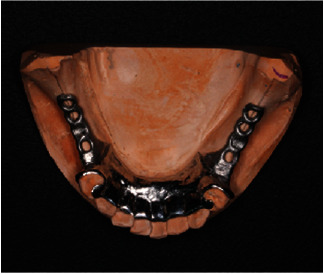
Chrome cobalt framework.

**Figure 18:**
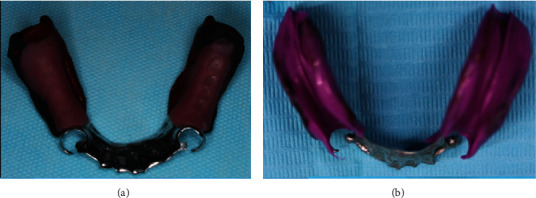
(a) Border molding on the distal free end saddle. (b) Final impression with medium body.

**Figure 19:**
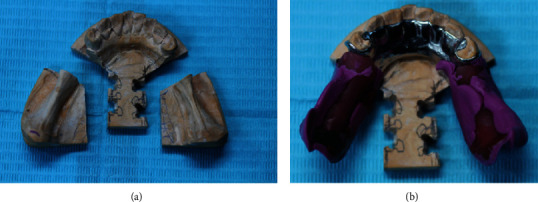
(a) Section the free end saddle on the master cast and create notches. (b) Attach the metal framework on the master cast.

**Figure 20:**
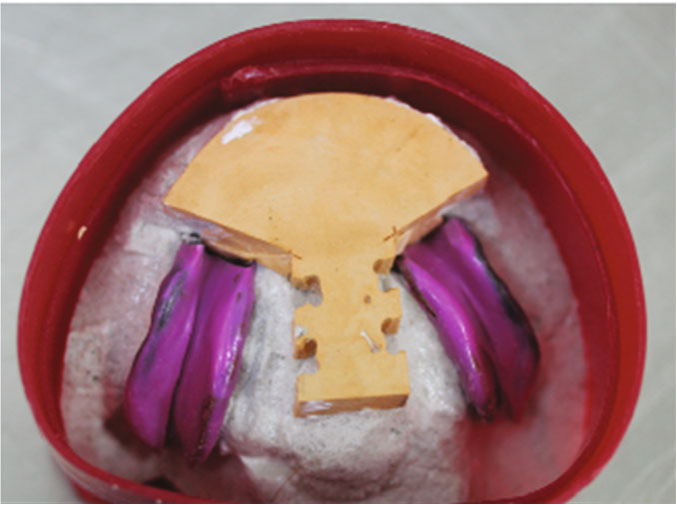
Boxing the impression.

**Figure 21:**
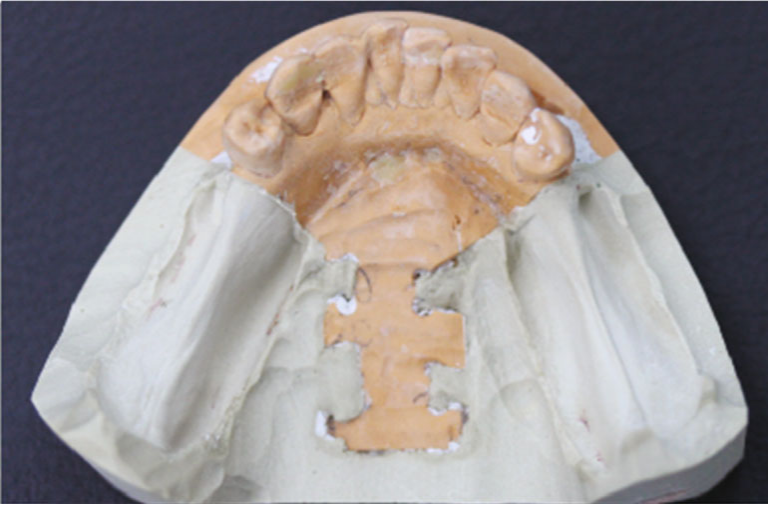
Altered cast.

**Figure 22:**
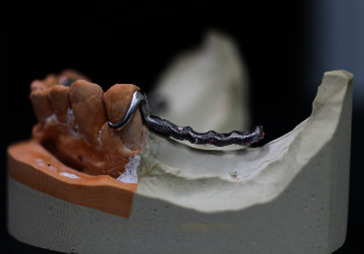
Metal framework on the altered cast.

Cingulum rest on (FDI 33 and 43) and mesial occlusal rest on (FDI 34) were prepared on the teeth. Lower impression was taken for fabrication of metal framework of lower chrome cobalt removable partial denture (Figure 15). Impressions were poured in the laboratory to fabricate the refractory cast with wax up of the chrome cobalt framework (Figure 16). Try-in the metal framework in the patient's mouth (Figure 17). A special tray on the distal free end saddle was fabricated, and impression was taken using border molding technique and monophase impression material (Figures 18(a) and 18(b)); then, altered cast technique was used for the free end saddle area to preserve the residual ridges, improve stress distribution, decrease food impaction, and decrease torqueing of abutment teeth. Old cast was sectioned on the distal end, and the new impression was reattached, as shown in Figures 19(a) and 19(b). Beading and boxing the cast was done, and stone was poured as shown in Figure 20, while Figure 21 shows the cast after the altered cast technique is done. Then, the framework was tried on the cast and was noticed that there is a space between the metal framework and the cast on the free end saddle area as shown in Figure 22.

## 7. Jaw Relation Records

**Figure 23:**
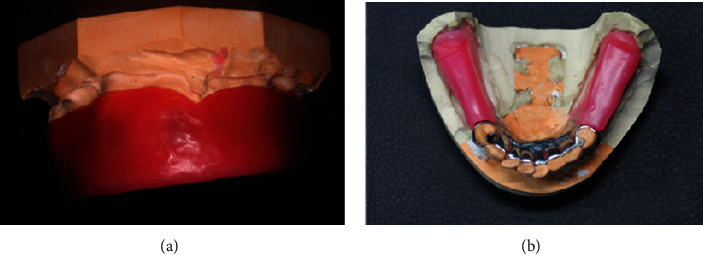
Occlusal rims for jaw relationship records.

**Figure 24:**
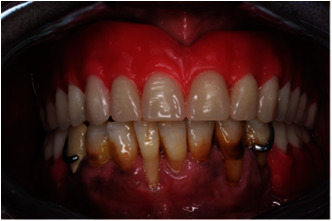
Teeth set up and try-in.

Once the upper secondary metal framework and the lower metal framework were ready, occlusal wax bite rims were fabricated (Figures 23(a) and 23(b)). Occlusal plan, smile line, dental midline, and canine line were adjusted and marked. Both vertical and horizontal jaw relationship records were taken in centric relation, and facebow transfer was recorded to mount the case on semi-adjustable articulator. Teeth set up try-in to check the occlusion, phonetics, and esthetics was done (Figure 24). Final processing of the removable prosthesis was done. For the occlusal schema, a bilateral balanced occlusion was utilized.

## 8. Delivery Stage

**Figure 25:**
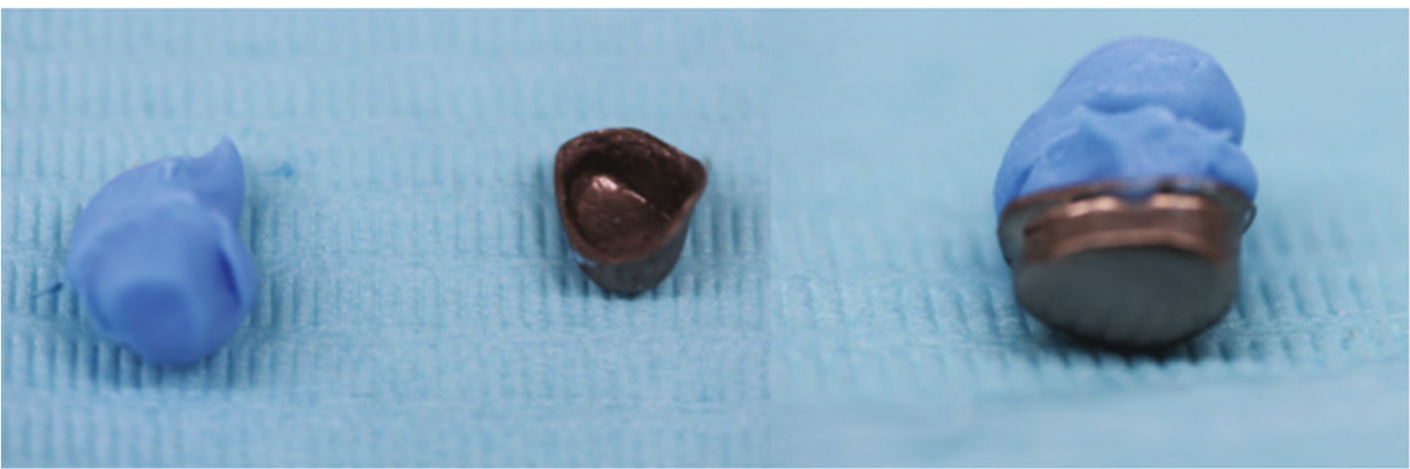
Index of the intaglio surface of the primary coping.

**Figure 26:**
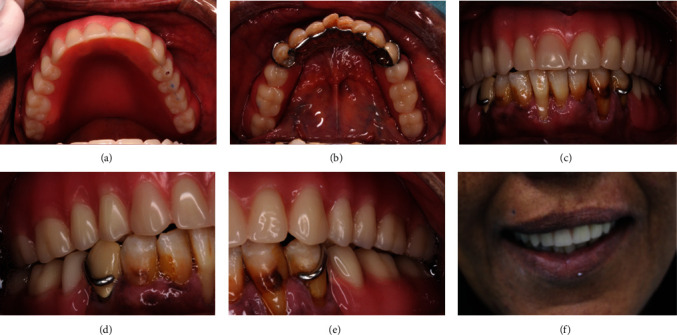
(a)–(f) Final prostheses upper telescopic complete denture and lower chrome cobalt removable partial denture.

The permanent cementation protocol for the primary copings was done one by one, while the superstructure was seated each time over all copings to ensure passive fitting. To avoid excess cement overflow, index of the intaglio surface of the primary coping was made using silicon bite registration material (Figure 25). The primary coping was loaded with the cement, and then, the index was placed to remove the excess cement and then was inserted on the tooth and light cured according to the manufacturer instruction. Upper telescopic complete denture and lower chrome cobalt removable partial prostheiss was delivered (Figures 26(a)–26(f)). Oral hygiene instructions and a three-month recall appointment were suggested.

## 9. Discussion

Oral health has been recognized as an integrated part of general health. People with multiple missing teeth has reported with masticatory function problems and poor nutritional status [16]. A survey on the relation between the missing teeth and the quality of life has shown that edentulous patient is having low quality of life, and their general health is affected when their teeth are not replaced due to functional and aesthetical reasons [17, 18].

Treatment modalities for partially edentulous patients range from removable prosthesis, fixed prosthesis, implant supported removable prosthesis, or implant supported fixed prosthesis. Clinical decision is based on the status of the abutment, periodontium, bone availability, and patient medical health and presences. Nowadays, dental implants have been popular treatment option for replacing missing teeth due to its high survival rates. The literature has reported high survival rate for implants. Zembic et al. (2014) have reported that a survival rate for single implant in 5 years was 97.6% with minimal biological and technical complications [19]. Muddugangadhar et al. (2015) reported that survival rate of implant tooth supported prostheses was 94.525% after 5 years in function [20]. In addition, the survival rate of implant supported removable partial dentures with distal extension was reported to be 91–100% with marginal bone loss ranging from 0.3 to 2.30 mm [21].

The residual alveolar ridge undergoes rapid bone loss in all dimensions after tooth loss, and it is well known and documented in the literature [22, 23]. This phenomenon is rapid and progressive, while bone is maintained around natural teeth and dental implants [23, 24]. Furthermore, complete or partial edentulism not only impairs the oral function but also affects the facial appearance and psychological condition of the patient [25].

In our case, the abutment teeth especially in the upper arch are weak to support fixed partial prosthesis and are considered unsuitable to support a removable partial prosthesis unlike the lower arch. Patient age and limited budget were taken into consideration by the author; hence, a telescopic complete denture was chosen as a favorable treatment option. Another alternative treatment option for our case could be implant supported fixed and/or removable prosthesis, yet due to patient preference to not have any surgical procedure, we decided that telescopic removable prosthesis is a good treatment plan option.

Telescopic denture is indicated when a few unfavorably distributed abutment teeth remained within the arch [26]. Langer and Langer (2000) and Dąbrowa et al. (2007) highlighted the advantage of telescopic crowns, such as axial loading of teeth and the abutment, reduced tilting forces on the abutment, easy oral hygiene maintenance, and ease for addition, adjustment, and repair [9, 27]. In this case, preserving the abutment teeth (FDI 16, 13, 11, and 26) provided proprioception for the patient, preserved the alveolar ridge, provided better masticatory function due to the presence of the periodontal proprioception, increased support, provided stability and retention.

Survival rate of telescopic retained removable prostheses in severely reduced dentition was 93.9% for abutment teeth and 87.5% for telescopes [28], whereas another study compared the survival rate between the telescopic removable prostheses and the conventional removable prostheses (clasp retained) and found no statistical difference [29]. However, crown decementation was significantly higher among the telescopic removable prosthesis 76.9% compared to conventional ones 28.3% [29]. A Longitudinal follow-up study of 5–10 years showed that telescopic prostheses has lower failure rate compared to clasp retained partial removable prosthesis [30]. In our case, the abutment teeth for the telescopic retainers if in future they were lost or extracted, the denture could still function without compromising the occlusion and aesthetic. Arnold et al. (2017) found that telescopic crowns with additional retentive elements had the highest retention forces [31]. Others found that using telescopic removable prostheses had improved the oral-health-related quality of life of patients [32].

## 10. Conclusion

Telescopic removable prosthesis has many advantages and disadvantages. Hence, dentists should be careful during treatment planning of partially edentulous patients. They have to carefully evaluate the remaining teeth and keep in their mind the higher cost and the long time needed for fabrication of the telescopic removable prostheses.

Clinical and laboratory steps have to be accurate. Sufficient tooth reduction is necessary; otherwise, prostheses will be over contoured. Furthermore, dental technician plays a major role in the success or failure of the prosthesis due to technical demands of the fabrication process of the telescopic removable prostheses.

This case report documents the clinical steps for fabrication and utilizing the telescopic removable prostheses to fully rehabilitate partially edentulous patient with conventional approach. The management of partially edentulous cases by minimal invasive approach was different in the UAE as conventional implant with bone graft placement is prevalent. The patient was very pleased with her improved aesthetic, function, and quality of life.
